# Lesion-specific 3D-printed moulds for image-guided tissue multi-sampling of ovarian tumours: A prospective pilot study

**DOI:** 10.3389/fonc.2023.1085874

**Published:** 2023-02-13

**Authors:** Maria Delgado-Ortet, Marika A. V. Reinius, Cathal McCague, Vlad Bura, Ramona Woitek, Leonardo Rundo, Andrew B. Gill, Marcel Gehrung, Stephan Ursprung, Helen Bolton, Krishnayan Haldar, Pubudu Pathiraja, James D. Brenton, Mireia Crispin-Ortuzar, Mercedes Jimenez-Linan, Lorena Escudero Sanchez, Evis Sala

**Affiliations:** ^1^ Department of Radiology, University of Cambridge, Cambridge, United Kingdom; ^2^ Cancer Research UK Cambridge Centre, Cambridge, United Kingdom; ^3^ Cancer Research UK Cambridge Institute, University of Cambridge, Cambridge, United Kingdom; ^4^ Cambridge University Hospitals NHS Foundation Trust, Cambridge, United Kingdom; ^5^ Department of Oncology, University of Cambridge, Cambridge, United Kingdom; ^6^ Department of Radiology, Clinical Emergency Children’s Hospital, Cluj-Napoca, Romania; ^7^ Research Center for Medical Image Analysis & Artificial Intelligence (MIAAI), Danube Private University, Krems, Austria; ^8^ Department of Information and Electrical Engineering and Applied Mathematics, University of Salerno, Fisciano, SA, Italy; ^9^ Dipartimento Diagnostica per Immagini, Radioterapia Oncologica ed Ematologia, Policlinico Universitario A. Gemelli IRCCS, Rome, Italy; ^10^ Dipartimento di Scienze Radiologiche ed Ematologiche, Università Cattolica del Sacro Cuore, Rome, Italy

**Keywords:** precision oncology, ovarian cancer, cancer imaging, radiogenomics, co-registration, 3D-printing, custom tumour moulds, tumour sampling

## Abstract

**Background:**

High-Grade Serous Ovarian Carcinoma (HGSOC) is the most prevalent and lethal subtype of ovarian cancer, but has a paucity of clinically-actionable biomarkers due to high degrees of multi-level heterogeneity. Radiogenomics markers have the potential to improve prediction of patient outcome and treatment response, but require accurate multimodal spatial registration between radiological imaging and histopathological tissue samples. Previously published co-registration work has not taken into account the anatomical, biological and clinical diversity of ovarian tumours.

**Methods:**

In this work, we developed a research pathway and an automated computational pipeline to produce lesion-specific three-dimensional (3D) printed moulds based on preoperative cross-sectional CT or MRI of pelvic lesions. Moulds were designed to allow tumour slicing in the anatomical axial plane to facilitate detailed spatial correlation of imaging and tissue-derived data. Code and design adaptations were made following each pilot case through an iterative refinement process.

**Results:**

Five patients with confirmed or suspected HGSOC who underwent debulking surgery between April and December 2021 were included in this prospective study. Tumour moulds were designed and 3D-printed for seven pelvic lesions, covering a range of tumour volumes (7 to 133 cm^3^) and compositions (cystic and solid proportions). The pilot cases informed innovations to improve specimen and subsequent slice orientation, through the use of 3D-printed tumour replicas and incorporation of a slice orientation slit in the mould design, respectively. The overall research pathway was compatible with implementation within the clinically determined timeframe and treatment pathway for each case, involving multidisciplinary clinical professionals from Radiology, Surgery, Oncology and Histopathology Departments.

**Conclusions:**

We developed and refined a computational pipeline that can model lesion-specific 3D-printed moulds from preoperative imaging for a variety of pelvic tumours. This framework can be used to guide comprehensive multi-sampling of tumour resection specimens.

## 1 Introduction

High-Grade Serous Ovarian Carcinoma (HGSOC) is the most prevalent and lethal subtype of ovarian cancer (1). While high levels of genomic complexity and clonal expansion are associated with poor outcome (2), comprehensive tissue multisampling to quantify cellular and molecular tumour heterogeneity is beyond the scope of current clinical diagnostic workflows, thereby limiting our understanding of the landscape of drug resistance mechanisms and potentially actionable targets in HGSOC.

Biomarkers that integrate routinely collected radiological data with molecular features may improve prediction of patient outcome and treatment response (3, 4). However, radiogenomic studies to date predominantly rely on retrospective cohorts and a single tumour sample from a single site per case – thus introducing an unquantified risk of sampling bias, and offering limited insight into the spatial relationship between radiomic features (5) and molecular heterogeneity at the whole-tumour level. This represents one major barrier to clinical implementation of radiogenomic biomarkers.

Biological validation of radiomic habitats –regions that share quantitative imaging characteristics (6, 7) – requires fine co-registration between tissue and imaging coordinates to allow systematic multi-sampling between as well as *within* radiomic habitats (8) emphasising the need for dedicated and improved co-registration methods. Patient-specific three-dimensional (3D) moulds have been proposed to allow co-registration between tumour tissue biopsies and preoperative imaging for subsequent multimodal data correlation and radiogenomic (6, 7) studies in several tumour-types, counting prostate (9–16), hepatic (17, 18) and renal (7, 19) cancers as well as a case report in ovarian cancer (6). These approaches represent important steps toward a detailed 3D spatial understanding of the wide relationship between molecular and radiomic heterogeneity.

In practice, however, two major factors limit wider implementation of 3D mould-based methods across institutions. First, most published works discuss only the end product and not the technical development process. Second, key anatomical, biological and clinical pathway-related aspects of ovarian tumour diagnosis and treatment present unique challenges to the implementation of previously proposed methods. For example, once adnexal tumours are resected, the orientation of those specimens for tissue sampling is often challenging due to the lack of anatomical landmarks. The purpose of this paper is therefore to provide a detailed, comprehensive illustration of the stages of development performed for our use case, to benefit future 3D mould-based work in the gynaecological cancer research community.

To generate a framework for 3D mould-based tumour sampling that can cater for the diversity of ovarian tumours encountered in clinical practice, we conducted a pilot study of five illustrative cases in their primary diagnostic phase. Through an adaptive process described in this work, we have developed and refined an automated computational pipeline that computes the shape and size of tumour delineated on routine computed tomography (CT) or magnetic resonance imaging (MRI) to inform lesion-specific mould printing prior to planned surgical resection. Our method is specifically aimed to allow for detailed spatial correspondence between imaging and tissue-derived data. Multidisciplinary work of this kind is inherently operationally complex – to maximise the reproducibility of our work, we provide a detailed overview of case selection and evaluation, computational modelling, tissue processing and critical considerations around the research pathway development.

## 2 Methods

### 2.1 Study design and patient cohort

Five patients with confirmed or suspected HGSOC undergoing debulking surgery between April and December 2021 at Addenbrooke’s Hospital (Cambridge University Hospital NHS Foundation Trust, Cambridge, UK) were enrolled in this prospective pilot study. Inclusion required informed written consent to the CTCR-OV04 observational study (Cambridgeshire and Hertfordshire Research Ethics Committee approval reference 08/H0306/61).

Representative cases of the two major treatment pathways in HGSOC (3), i.e. immediate (IPS) and delayed primary surgery (DPS), were included to explore unique challenges associated with each. Inclusion of DPS cases required prior histopathological confirmation of HGSOC. IPS procedures are often performed both as a diagnostic and therapeutic intervention if prior laparoscopic biopsy had not been indicated or possible. The IPS cases without prior histological diagnosis were selected on the basis of clinical suspicion for HGSOC due to significantly elevated serum CA125 (> 4 times upper limit of normal at 35 units per millilitre) and CT imaging features highly suspicious for HGSOC assessed by a Consultant Radiologist with special expertise in gynaecologic oncological imaging (RW/ES).

### 2.2 Imaging review and segmentation

Preoperative CT and MRI scans were anonymised prior to downloading from the hospital PACS system. This included removal of all directly identifiable information from the images themselves, and deletion of all directly identifiable information from the DICOM headers in the accompanying image metadata. De-identified images were then downloaded in DICOM format from the hospital PACS system and separated into different series using OsiriX DICOM Viewer (version 12.0, Pixmeo SARL, Geneva, CH).

**Figure 1:**
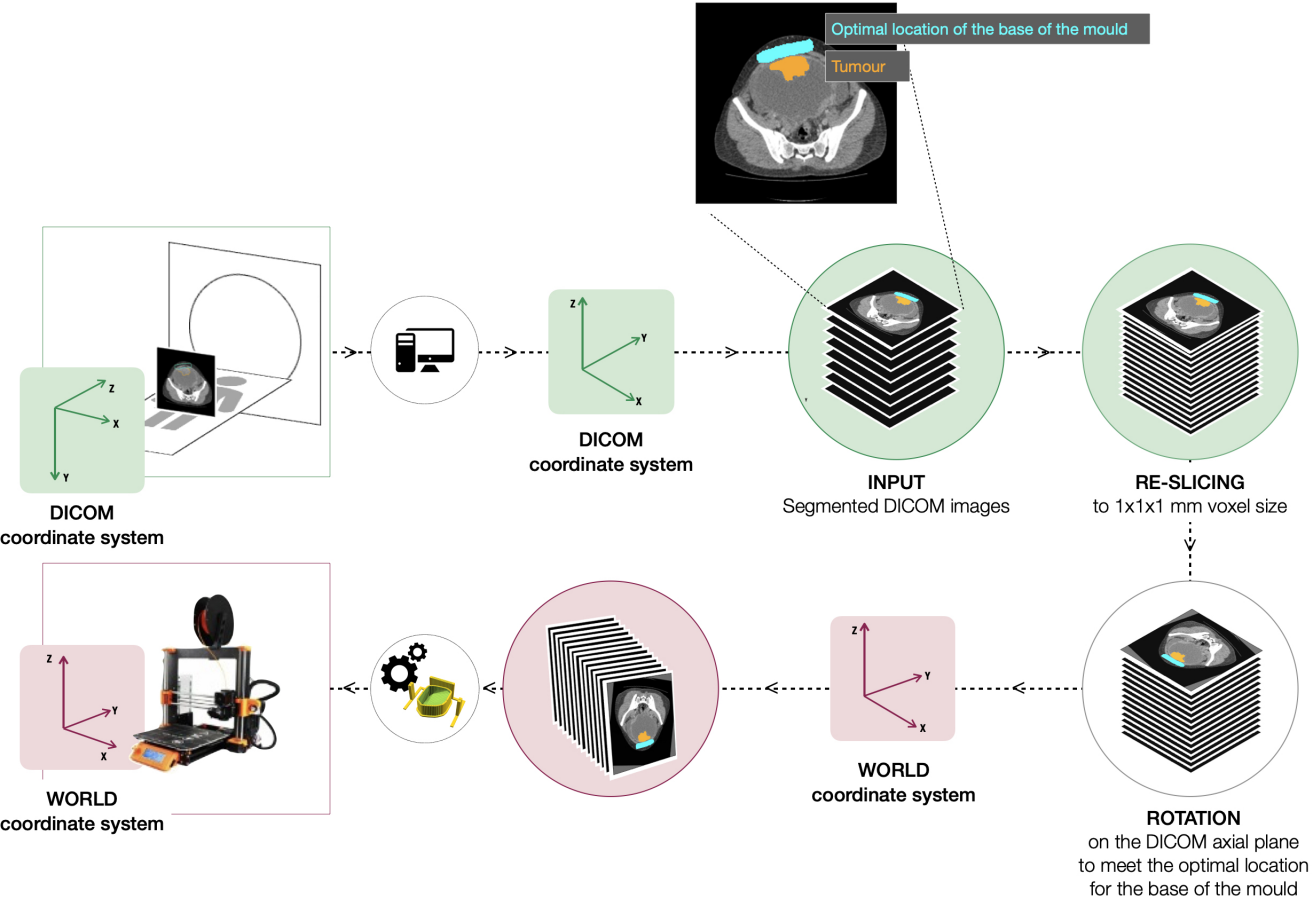
Initial steps of the pipeline to re-slice and re-orientate the segmentation to build the mould complying with the imposed base of the mould location (manually segmented, in cyan) and the slicing direction (in the anatomical axial plane). When transforming the image to the world coordinate system (WCS), the slicing direction is perpendicular to the x axis.

Manual segmentations were performed by a Radiologist in training (CM: 3 years’ experience; VB: 6 years’ experience) using Microsoft Advanced Medical Image Labeler (version 1.0.0.0, project InnerEye, Microsoft, Redmond, WA, USA) or the Open Health Imaging Foundation viewer (Open Health Imaging Foundation, Massachusetts Institute of Technology, Cambridge, MA, USA, version 3.2.0) *via* its plugin to XNAT, hosted at the local node of the repository established by the CRUK National Cancer Imaging Translational Accelerator (NCITA, https://ncita.org.uk) (20). Challenging segmentations were verified by a board certified Gynaecological Radiologist (RW: 8 years’ experience as Consultant Radiologist). Pelvic lesions representing confirmed or suspected tumour were segmented as the region(s) of interest (ROIs) in each case. To automate the tumour rotation steps of the computational pipeline (see Section 2.3), the optimal location of the base of the mould (the surface where the tumour will sit on) for the final two cases was added to the manual segmentations by the radiologist as an extra ROI (**Figure 1**). Optimal base positioning was selected upon tumour shape and composition on imaging to maximise specimen stability within the mould for increased slicing accuracy, prioritising smooth and solid tumoural surfaces to be on the lowest portion.

### 2.3 Computational pipeline

A computational pipeline was implemented to generate a mould to specifically hold and slice the segmented lesion in the axial plane, using the input DICOM images and the DICOM-RT file containing the segmented ROIs. A series of tunable values (e.g. slice thickness for tumour dissection, mould height) allows the user to customise the final design to case-specific requirements. The pipeline runs on Python with an interface to OpenSCAD for building the mould structures. All code is available on https://github.com/mariadeor/3d-moulds-for-ovarian-cancer.

#### 2.3.1 Tumour rotation

The first step of the pipeline re-slices and interpolates the input images to a standardised isotropic voxel size of 1×1×1 mm using zero-order spline interpolation to homogenise the process independently of the reconstructed slice thickness of the scans. From there, the tumour segmentation is orientated such that it complies with (a) the imposed base of the mould location and (b) the slicing direction, in the anatomical axial plane (**Figure 1**). During step (a), the segmentation mask is rotated on the DICOM axial plane to ensure the tumour region adjacent to the segmented optimal location for the base of the mould sits at the bottom. This step could only be automated after the additional ROI outlining the positioning of the base of the mould was added to the input manual segmentation. Step (b) involves the transformation from the DICOM coordinate system to the world coordinate system (WCS), fixed in relation to the print bed.

#### 2.3.2 Tumour modelling

**Figure 2:**
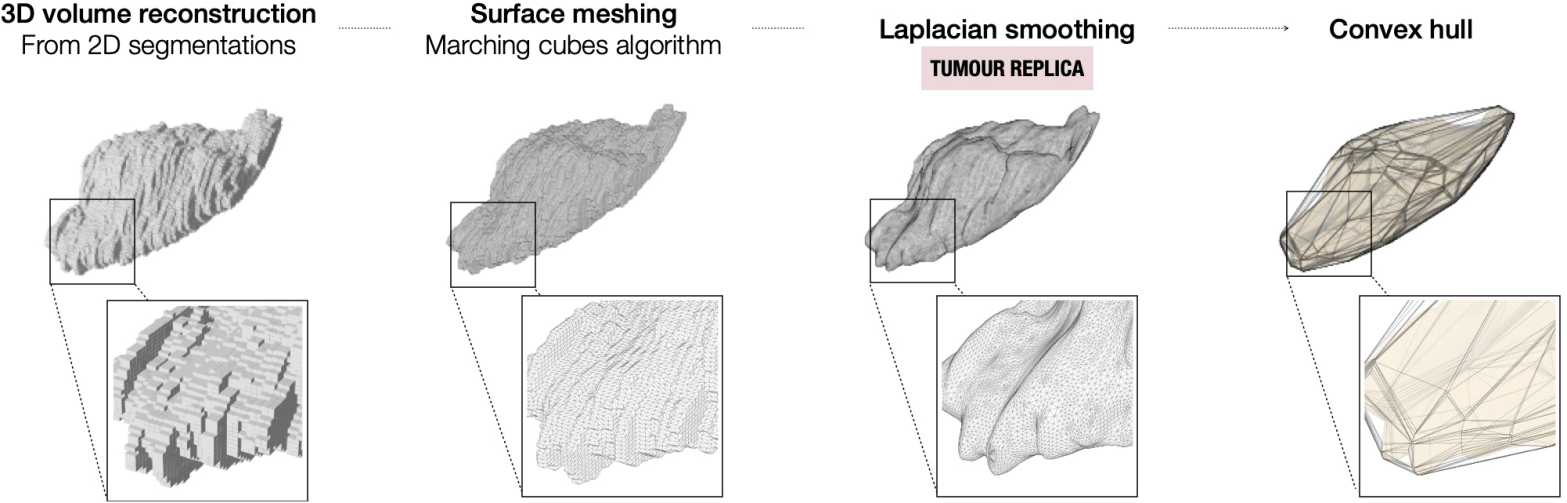
Steps for the modelling of the tumour from the 2D segmentations on the preoperative imaging.

Once the tumour segmentation is appropriately oriented in the WCS, the 3D tumour volume is reconstructed from the 2D segmentations. To minimise the layered appearance of the stack of 2D segmentations and increase the resemblance to the actual specimen, two algorithms are sequentially applied. First, the 3D surface mesh of the tumour volume is extracted using the Lorensen and Cline marching cubes algorithm (21). Next, the mesh is smoothed using Laplacian smoothing (λ = 1, defined empirically to ensure the smoothing of the tumoural volume while avoiding excessive shrinkage) (22, 23) (**Figure 2**). The resulting volume (tumour replica) is 3D-printed for improved orientation and visual assessment purposes.

#### 2.3.3 Mould modelling

**Figure 3:**
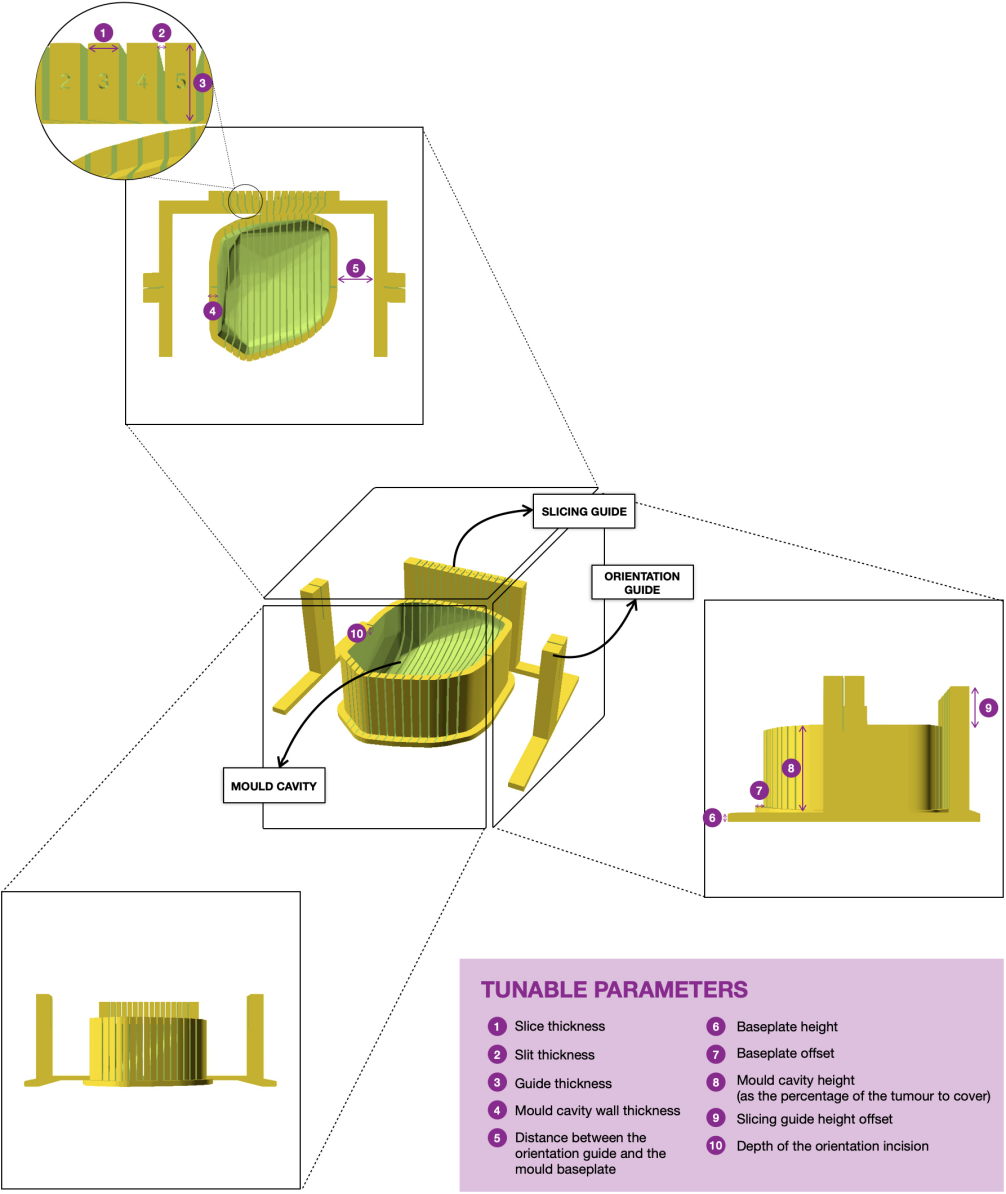
3D rendering of the final version of a mould. The tumour sits on the mould cavity and it is sliced by passing a knife through each of the slits of the slicing guide. Two single-slit guide walls are built parallel to the slicing direction for the slitting of the orientation line at the top of the specimen. The orientation guide determines the position of the orientation incision, which facilitates the correct orientation of the slices when removed from the mould. The mould is customisable and the tunable parameters for mould design are in purple.

The mould is designed to aid tumour slicing in the axial plane, which is the standard view for radiological assessment of the abdomen and pelvis. The final mould is an ensemble of three structures: (i) the mould cavity, (ii) the slicing guide and (iii) the orientation guide (**Figure 3**). Baseline structural design choices (open mould cavity and slicing guide on a single side) were based upon previous optimisation for renal cancers (7) and adapted to the challenges uniquely posed by pelvic lesions throughout this work. A single-sided mould (open cavity) was preferred as changes in the upper side (e.g. cyst rupture, specimen deformation due to the gravity effect on change of orientation) do not affect the accuracy and fitting of the specimen to the mould. Additionally, it requires shorter printing times, especially relevant for the integration of the research pathway in the clinical setting.

First, the cavity of the mould is carved around the convex hull of the tumour replica (**Figure 2**) and its height is defined through a tunable parameter specifying the percentage of tumour height to cover (**Figure 3**).

Next, the slicing guide is modelled: it covers the whole length of the tumour on the x axis (the equivalent on the WCS to the patient’s craniocaudal axis) and it has the number of slicing slits resultant from dividing the tumour length by the slice width (tunable). The central slicing slit is aligned with the centre of the modelled tumour, and further slicing slits are placed at set intervals on either side of this. The slicing slits have tunable width and are projected to the mould cavity in order to guide the tissue knife all the way through the tumour upon slicing. In parallel to this process, the slicing slits positions are used to extract the 2D outlines of each tissue slice based on the segmentation. These can be printed in real size, have overlaid the base of the mould and the position of the “orientation incision” –explained below– and are fundamental for allowing the co-registration between the tissue and the imaging slices (See Results, Case 5).

The last structures to be built are the two orientation guides, which are single-slit guide walls located perpendicularly to the x axis at each end of the tumour. They have the purpose of determining the course of the “orientation incision” across the top part of the tumour, to ensure the correct orientation of the slices when removed from the mould. This is key for the registration of histopathological samples with imaging (see Results, Cases 3-4).

Finally, the structures are assembled onto a common baseplate. In order not to increase the printing time and minimise the waste of printing material, the baseplate is not a solid block but a tunable offset to the mould cavity and the walls attached.

### 2.4 3D-printing

All moulds and tumour replicas were 3D-printed using the Prusa i3 MMU2S MK3S (Prusa Research, Prague, Czech Republic) printer loaded with PLA filament. Preceding 3D model slicing and print preparation was done with PrusaSlicer software (PrusaSlicer version 2.3.1, Prusa Research, Prague, Czech Republic), setting the infill value to 20% and the layer height to 0.3 mm.

### 2.5 Intra- and post-operative workflow

Upon resection by a Specialist Gynae-Oncology Surgeon (HB, KH, PB), tissue specimens were immediately checked visually against their respective tumour replicas and placed within their respective moulds in the operating theatre. As an additional layer of confirmation of correct orientation, orientation sutures were placed by the surgeon prior to resection for the third and subsequent cases. Following confirmation of stability, specimen-bearing moulds were placed in designated clinical specimen containers and transferred to the Cambridge Human Research Tissue Bank (HRTB) following routine tissue transfer procedures.

At the HRTB, all specimens were reviewed and processed by a Consultant Histopathologist specialising in gynaecological cancers (MJ-L) according to standard procedures. Tumours were sliced within their corresponding moulds. Frozen sections were taken for cellularity assessment or to evaluate or confirm likely diagnosis in suspected or confirmed HGSOC, respectively. Benign pathology precluded further tissue processing for research purposes. Malignant tissue sampling for research use was restricted to tissue regions not required for diagnostic purposes in the patient’s clinical care.

## 3 Results

### 3.1 Clinical characteristics

Five patients were included in this pilot study (**Table 1**). Mean and median age were 53.8 and 54, respectively, with a range of 47 years between the eldest and youngest patient. Two IPS cases were found to have benign tumours following histopathological review of the IPS resection specimens, while the third IPS case was confirmed to have non-serous high grade pathology. Both DPS cases were treated with four cycles of neoadjuvant carboplatin paclitaxel chemotherapy prior to surgery for HGSOC confirmed on initial diagnostic biopsy. The time interval between imaging and surgery ranged from nine to 53 days (mean: 32.2, median: 40) reflecting individual clinical factors and health system pressures during the COVID-19 pandemic.

**Table 1:** Clinical characteristics of the pilot cohort.

Case	1	2	3	4	5
Prior histology	HGSOC	NA	NA	Insufficient sample but invasive component noted	HGSOC
FIGO stage	IIIC	NA	NA	IIB	IIIB
Surgery type	DPS	IPS	IPS	IPS	DPS
Disease laterality/distribution	Unilateral (left ovary) + peritoneal	Unilateral (right ovary)	Bilateral	Multifocal	Bilateral
Tumour characteristics	Cystic with limited solid component	Solid	Solid	Large cyst with large solid component	Solid
Final histology	HGSOC	Benign	Benign	High grade endometrioid	HGSOC

### 3.2 Image segmentation

For two cases undergoing neoadjuvant chemotherapy (NACT) following histopathological confirmation of HGSOC (Cases 1 and 5), routine NACT response assessment images were used for segmenting tumour regions corresponding to expected specimens from DPS. For the remaining three cases undergoing IPS, baseline diagnostic imaging was used.

**Figure 4:**
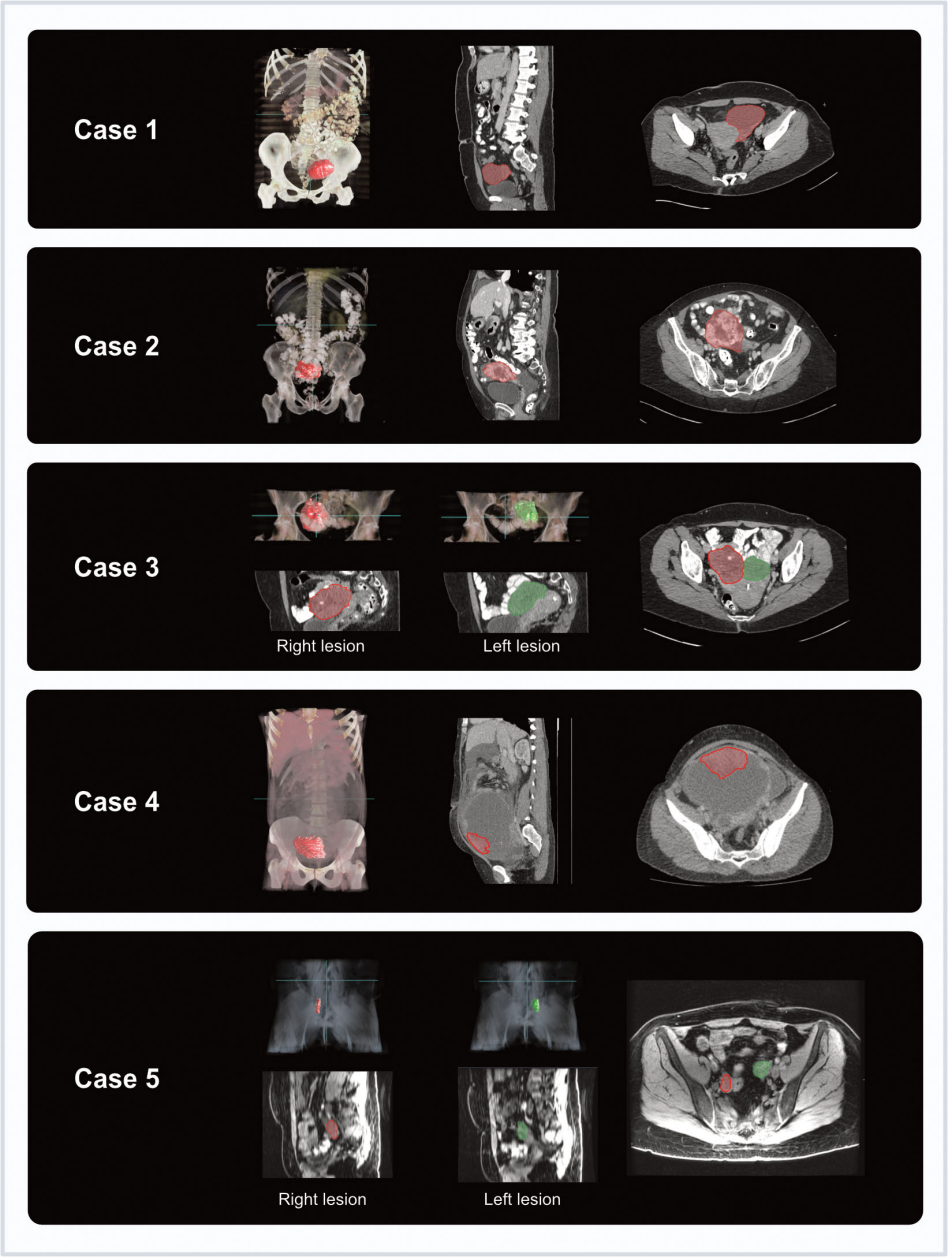
Coronal and axial imaging with overlaid tumour segmentations for each case (3D volume rendering from Microsoft Advanced Medical Image Labeler).

Tumours were delineated using CT for the majority of cases (Cases 1-4, six moulds). For one patient (Case 5, two moulds) who underwent CT for NACT response assessment, as well as additional MRI prior to surgery, the MRI was used as it was acquired closer to the time of surgery (nine days for MRI, 30 days for CT) (**Figure 4**). Relevant imaging acquisition parameters are summarised in **Supplementary Tables 1**, **2**.

Segmentations were performed between two and six days before surgery (median five days prior). Segmentation times varied between cases depending on complexity, taking up to one hour for particularly complex cases.

### 3.3 Mould generation and refinement

Eight lesion-specific moulds were designed and 3D-printed for a total of seven lesions, covering a range of tumour volumes (7 to 133 cm^3^) and compositions (cystic and solid proportions) (**Table 2**). Tumour replicas were also 3D-printed to visually confirm accurate representation of the shapes and sizes of the resected specimens (accurate in six out of seven lesions).

**Table 2:** Mould and pathway characteristics.

Case	1	2	3	4	5
Mould site(s)	Left ovary	Left ovary	Left ovary Right ovary	Anterior inferior solid mass within cystic lesion (two moulds covering the interior and exterior surface)	Left ovary Right ovary
Tumour volume (cm^3^)	125.6	132.9	Left: 69.1 Right: 104.8	Solid mass: 129.8	Left: 10.2 Right: 7.0
Imaging modality	CT	CT	CT	CT	MRI
Time between imaging and surgery	40 days	43 days	15 days	53 days	9 days
Time from study inclusion to surgery	6 days	3 days	3 days	8 days	16 days
Time from image segmentation to surgery	5 days	3 days	3 days	8 days	16 days
Mould 3D-printing time [hours:minutes]	04:30	04:56	Left: 04:13 Right: 04:52	Internal: 10:02 External: 10:30	Left: 02:36 Right: 02:16
Tumour replica 3D-printing time [hours:minutes]	02:36	02:54	Left: 02:12 Right: 02:32	Mass: 06:49	Left: 00:35 Right: 00:32

The computational pipeline for mould design took under two minutes to run in each case. 3D-printing took a median of 4 hours and 41 minutes (range 2-10 h) for the lesion-specific moulds and 2 hours and 32 minutes for the tumour replicas (32 min-7 h).

#### 3.3.1 Case 1

The main aims for the first case were: (a) to build a local research pathway that can be implemented without altering existing standard of care pathways (see Section 3.4); (b) to determine which anatomical structures to include in the mould design; and (c) to test if the meshing and smoothing of the image segmentations produced moulds and tumour replicas that matched the actual size and shape of the resected specimen.

A post-NACT HGSOC case with a sufficiently large lesion to span at least three specimen slicing positions (with a slice width of 10 mm) was selected. Specimen collapse following tissue slicing was expected due to the cystic nature of the lesion of interest, and sufficient tissue for sampling was not anticipated based on imaging features. Instead, overall mould design alone (rather than sampling) was the focus of this first pilot case.

**Figure 5:**
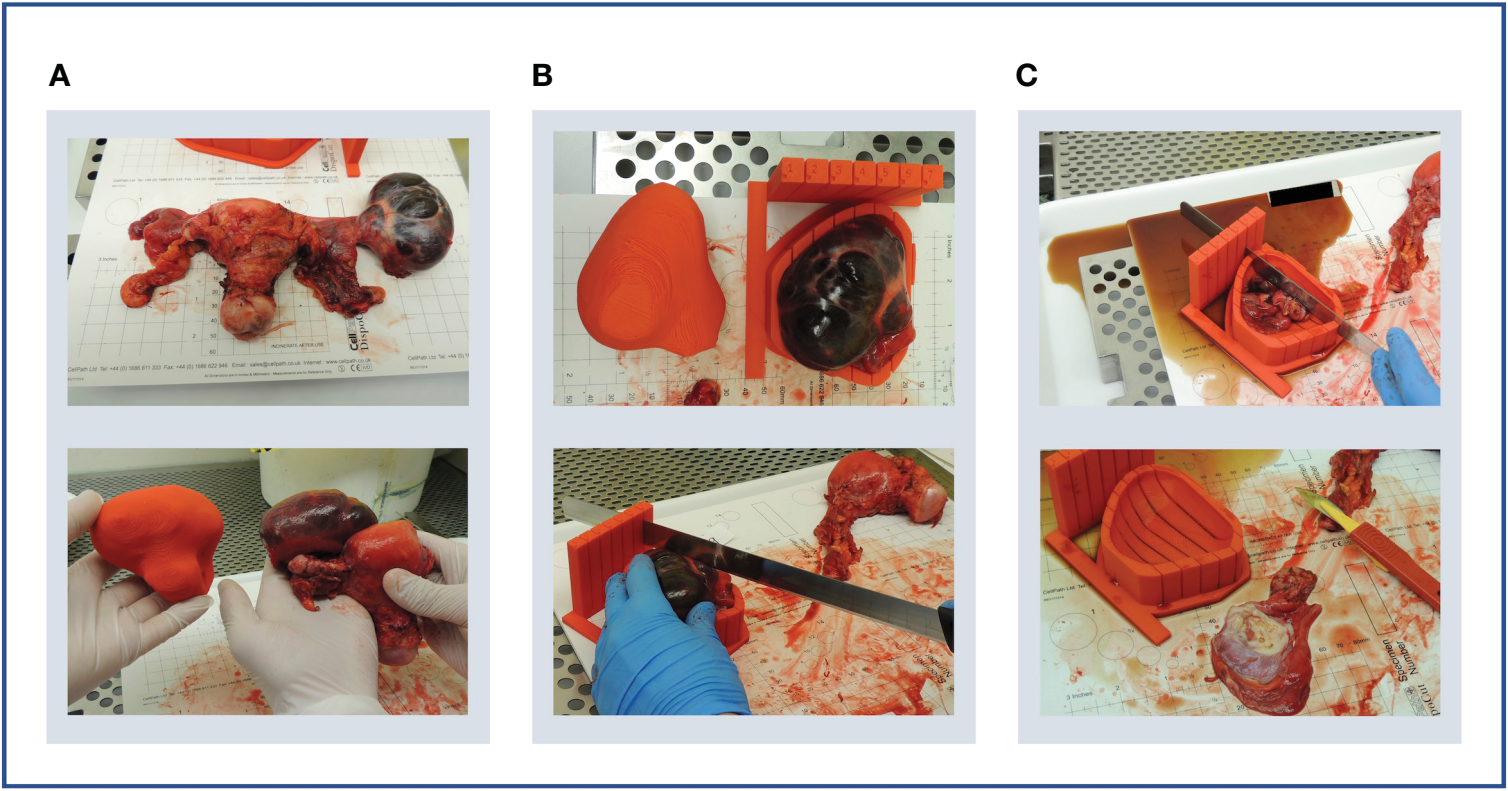
Case 1. **(A)** Upper panel: en bloc resection specimen. Lower panel: en bloc resection specimen with left ovarian tumour next to its 3D-printed tumour replica. **(B)** Upper panel: left ovarian tumour in its 3D mould, next to its tumour replica. Lower panel: specimen knife placed in slit prior to slicing. The 5 mm slits were too narrow for the knife. **(C)** Upper panel: the cystic tumour collapsed immediately on capsule rupture as expected, releasing cystic fluid. Lower panel: Tumour tissue was insufficient for research sampling as expected based on the pre-operative CT imaging.

A lesion-specific (left ovary only) mould was 3D-printed and used (an en bloc mould design which was trialled and deemed unsuitable as detailed in **Supplementary Material**). Given the lack of anatomical landmarks for orientation, the 3D-printed tumour replica was key in facilitating rapid specimen orientation once the tumour was detached from surrounding structures (**Figure 5A**). Confirming tumour orientation within the mould using the tumour replica also minimised subsequent manipulation of the specimen and the associated risk of cyst rupture. The replica allowed visual confirmation that the modelled volume accurately resembled the actual shape and size of the resected specimen (**Figure 5B**).

Although the specimen was stable in the mould, slicing resulted in the release of cystic fluid, causing the mass to collapse as expected (**Figure 5C**). Histopathological review confirmed the lack of sufficient tumour tissue for sampling, as anticipated based on pre-operative CT imaging. However, the test slicing proved useful in highlighting the need for the 0.5 mm knife slots to be widened to accommodate the full depth of the tissue knife.

The smooth symmetric tumour contour, in addition to the lack of anatomical landmarks, made the orientation of the meshed and smoothed tumour replica particularly challenging. While the position of the optimal base of the mould was agreed upon multidisciplinary discussion, it was not included in the segmentation which would have allowed automated processing. Consequently, the computational pipeline required manual input for tumour rotation, a step that could potentially be automated by adding such annotation as shown in later iterations (Cases 4 and 5). Despite the fact that manual rotation on the axial plane was successful, the subsequent transformation to the WCS was unsuccessful, implying that the slicing axis was not aligned to the axial plane. This highlighted the importance of ensuring accurate transformation to the WCS as per enabling specimen slicing along the desired anatomical plane.

#### 3.3.2 Case 2

To test specimen slicing and slice orientation, a suspected malignant right ovarian tumour for IPS resection was selected on the basis of predominantly solid tumour composition. While frozen section assessment and subsequent review of diagnostic formalin-fixed paraffin-embedded (FFPE) blocks confirmed benign tumour pathology and thereby precluded tissue analysis for research purposes, the case provided key learning to improve the mould creation process.

**Figure 6:**
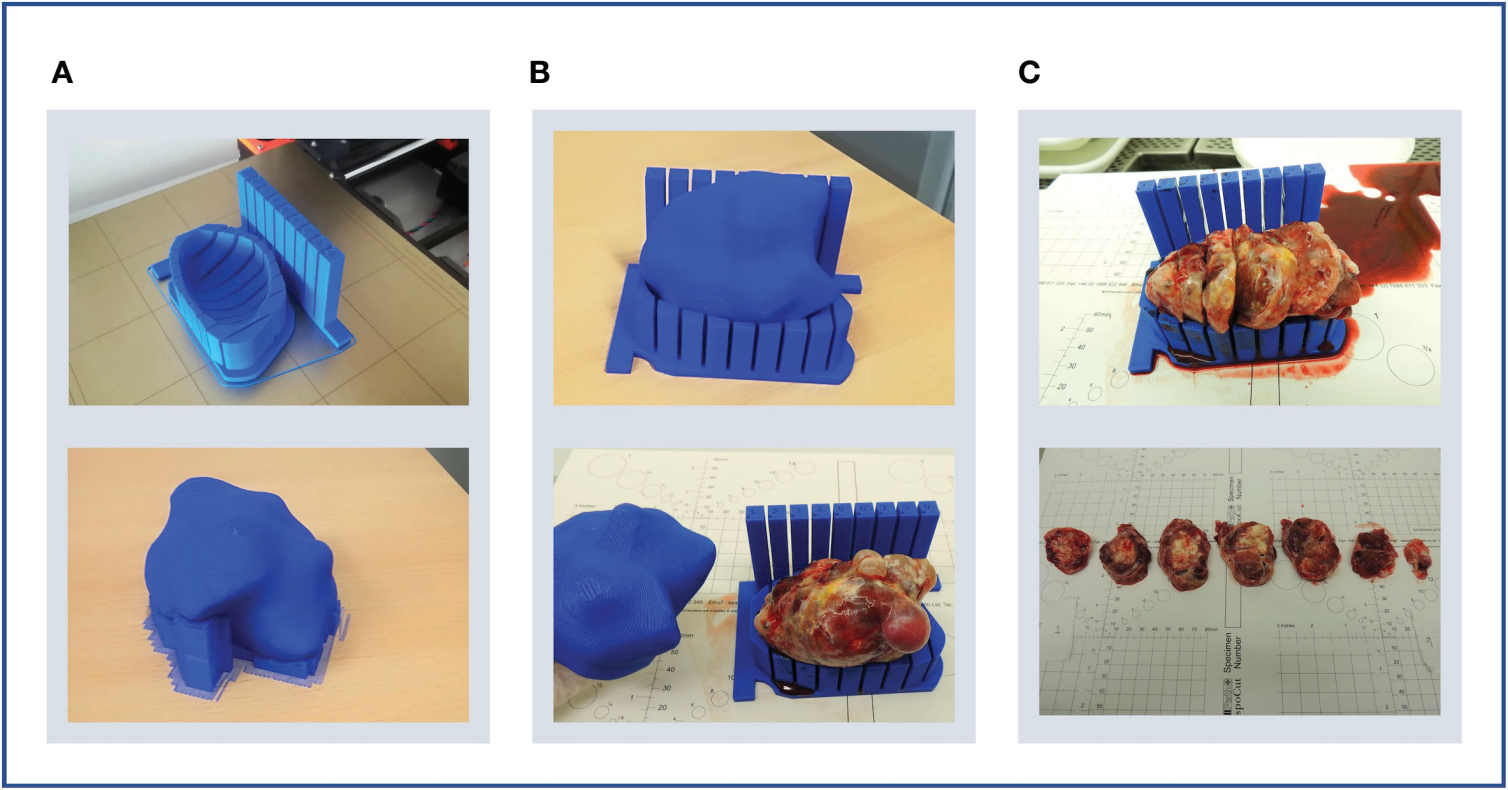
Case 2. **(A)** Upper panel: lesion-specific mould on the print bed. Determining tumour specimen placement based on the shape of the mould cavity alone is challenging, hence a tumour replica is also printed (lower panel). A temporary scaffolding to support the emerging replica during printing is added and easily removed when printing is complete. **(B)** Upper panel: the tumour replica fits the mould closely as the mould and replica are both printed based on the same segmentation. Lower panel: the resection specimen is placed directly into its correct position based on the placement of the replica. **(C)** Upper panel: the tumour was successfully sliced, but the shallow tumour cavity led to tissue slices overspilling. Lower panel: even with a tumour with macroscopically visible substructures, slice orientation in relation to the mould and to each other is lost on retrieval from the mould.

We successfully incorporated our learning from Case 1, including: specimen orientation using the tumour replica, which closely resembled the resection specimen (**Figures 6A, B**), and tumour slicing through the entire lesion diameter in every slit following increased knife slit width to 1 mm (**Figure 6C**).

Three key mould design challenges were highlighted by this case. Firstly, insufficient depth and thereby lateral support produced slices that spilled over the mould edge. This led to decreasingly accurate slicing with each cut due to lateral slice movement (**Figure 6C**). This resulted from setting the maximum mould cavity height to the widest xy dimension of the tumour in the original version of the pipeline –the rationale being that increasing the mould wall height upwards to the level of a narrower point would constrict the mould brim diameter and impede specimen placement. Secondly, tissue retrieval from the mould after slicing highlighted that slice orientation is easily lost without a method to mark slice orientation in relation to each other and to the mould whilst inside the mould (**Figure 6C**). Finally, this case also highlighted the need for accurate orientation to WCS as in Case 1.

#### 3.3.3 Case 3

A further case with solid ovarian disease was chosen to address mould design improvements indicated by Case 2.

**Figure 7:**
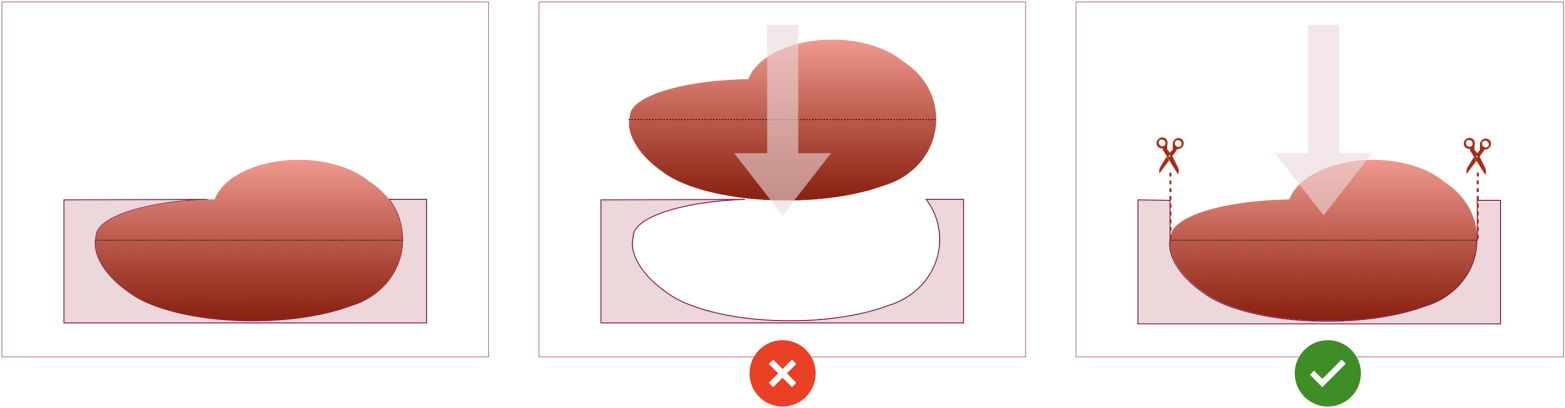
Schematic of the design of the mould cavity for cases in which the height of the cavity is greater than the height where the widest surface of the tumour occurs.

**Figure 8:**
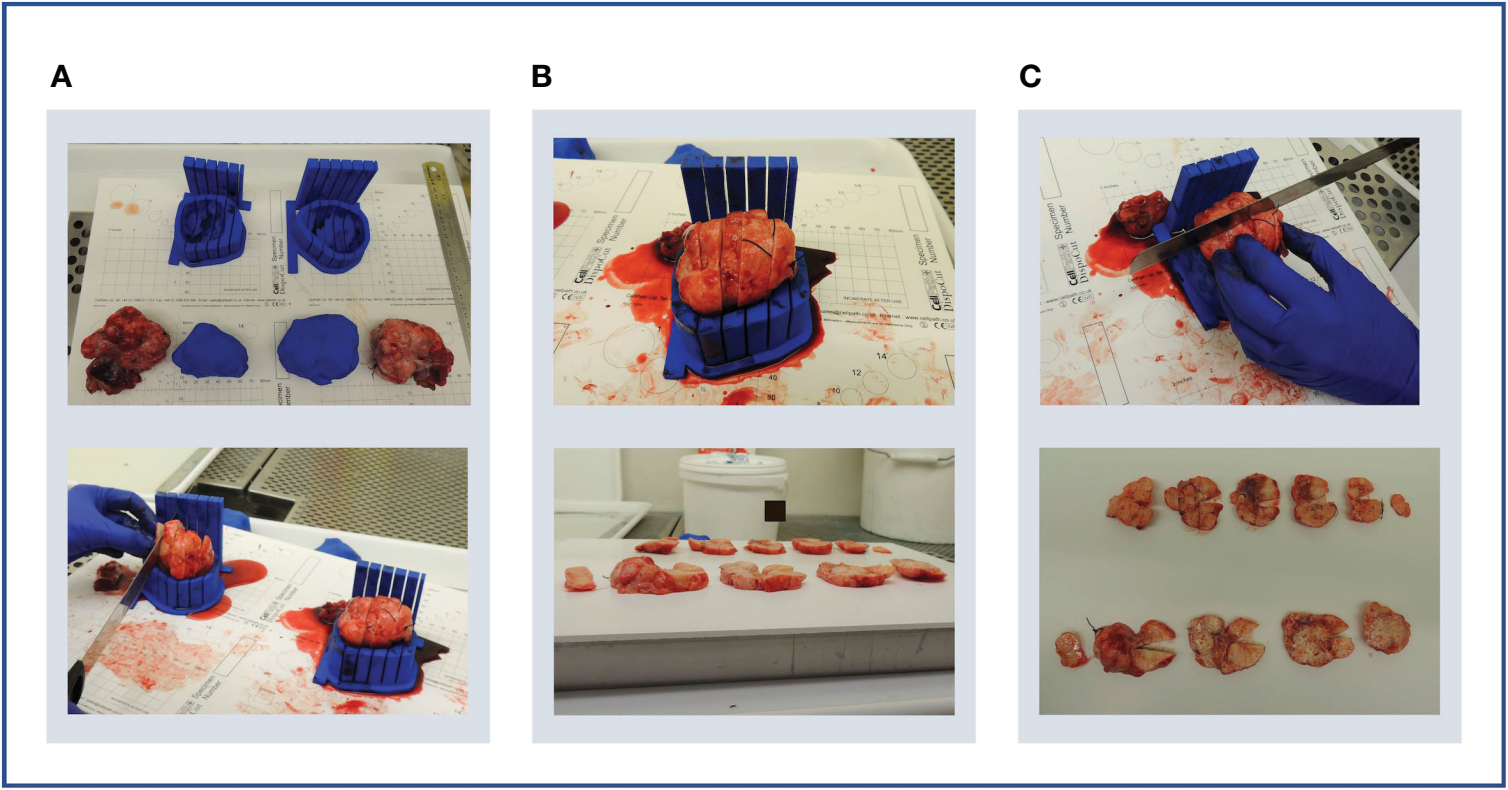
Case 3. **(A)** Upper panel: bilateral specimens with respective moulds and replicas. Lower panel: both specimens were sliced within their respective moulds. **(B)** Upper panel: due to shallow mould cavities and slice overspill, accurate cutting into 10 mm slices was challenging, and resulted in unequal slice thickness (lower panel). **(C)** Upper panel: an orientation cut was trialled to mark slice orientation. Lower panel: the orientation cut keeps track of slice orientation to each other.

Firstly, we tested whether the problem with insufficient mould height was an isolated or recurrent problem. When mould cavity height was set to the maximum allowed by the original pipeline algorithm (i.e. the widest xy slice), similarly to the case above, the slice overspill issue recurred. This demonstrated that the mould height would need to be increased perpendicularly upwards (rather than following the narrowing tumour contour) from the widest xy dimension of the tumour (**Figure 7**) to avoid overspilling (**Figure 8B**).

**Figure 9:**
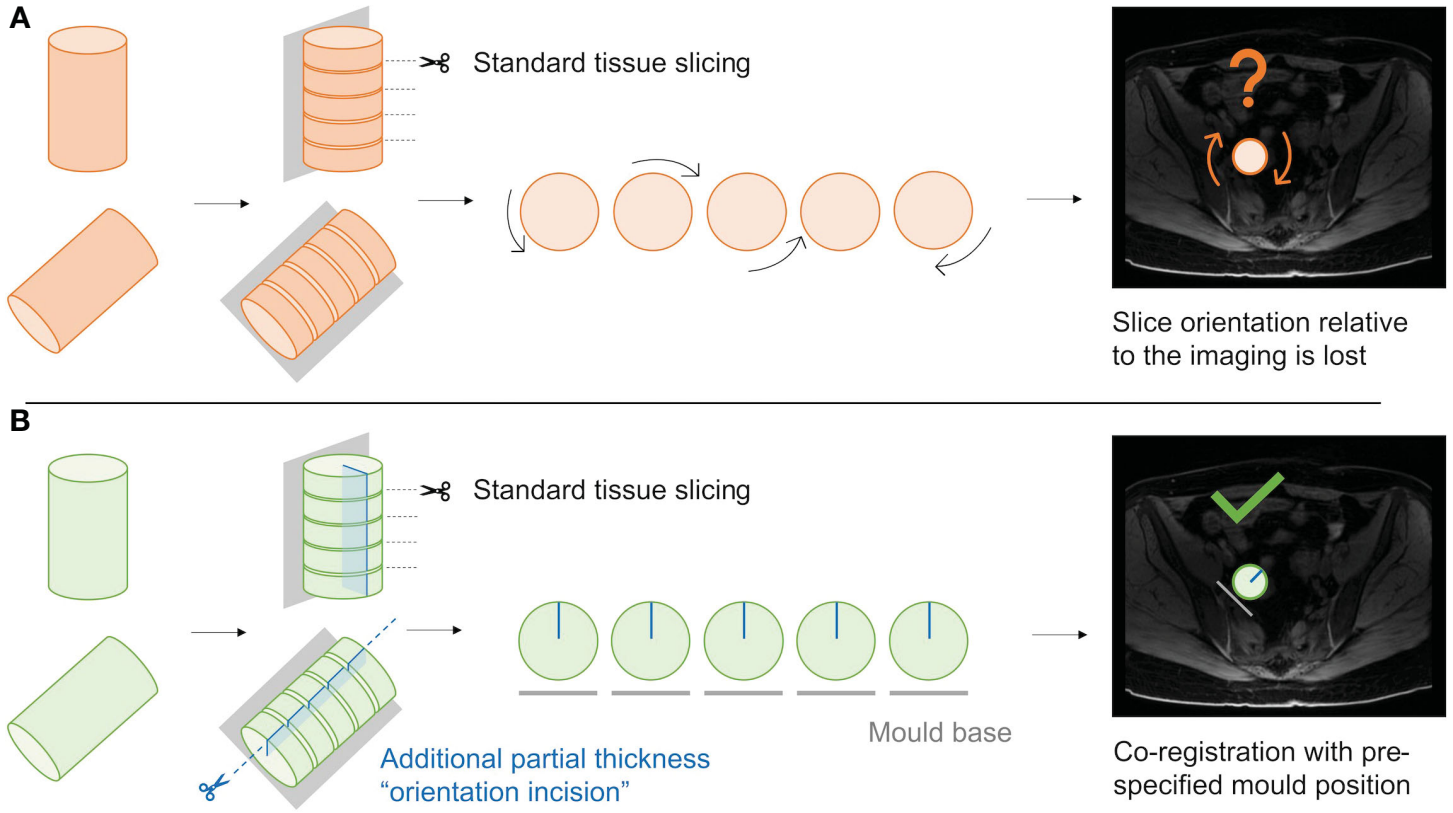
Schematic demonstrating the utility of the “orientation incision”. **(A)**; Without an orientation incision, any rotation of the tissue slices when removed from the mould cannot be quantified, and co-registration if radiological imaging is lost. **(B)**; a partial-thickness orientation incision across tissue slices allows orientation of slices to each other, and to their corresponding coordinates on the radiological imaging.

Secondly, the loss of slice orientation in relation to each other on removal from the mould was addressed by placing a partial-depth perpendicular incision across the tissue slices while remaining in the mould – we refer to this as the “orientation incision” (**Figure 8C**). This simultaneously placed a notch in each slice, serving as a physical mark that prevents inadvertent tissue slice rotation on removal from the mould. Additionally, if the position and depth of the orientation incision is known and dictated by the mould, it can also be used as a physical landmark for precise image-tissue co-registration. To achieve this, the orientation guide (**Figure 3**) was added to the mould structure to aid the slitting of the orientation incision, ensuring that it is both centred to the specimen and at a suitable depth to place a partial notch in the tissue slices without bisecting them (**Figure 9**).

Thirdly, accurate rotation and transformation to the WCS to orient the mould in relation to the intended slice alignment (axial plane) was once again hampered by the lack of anatomical landmarks or distinctive tumour shape features. An extra ROI drawn during segmentation, marking the optimal base location, was planned for the following case to automate accurate tumour rotation and transformation to WCS.

Additionally, this case presented an opportunity to test pathway compatibility with bilateral disease, with doubled modelling and 3D-printing time. The two moulds and replicas were completed in time for the planned surgery, and tumour replicas closely resembled the corresponding resection specimens as with previous cases (**Figure 8A**).

#### 3.3.4 Case 4

Cases 2 and 3 highlighted a challenge in terms of being unable to perform tissue sampling when applying this pathway to IPS cases which are only found to be benign at the time of frozen section post-operatively. Given this, a case with confirmed high grade malignant features on biopsy was selected next.

**Figure 10:**
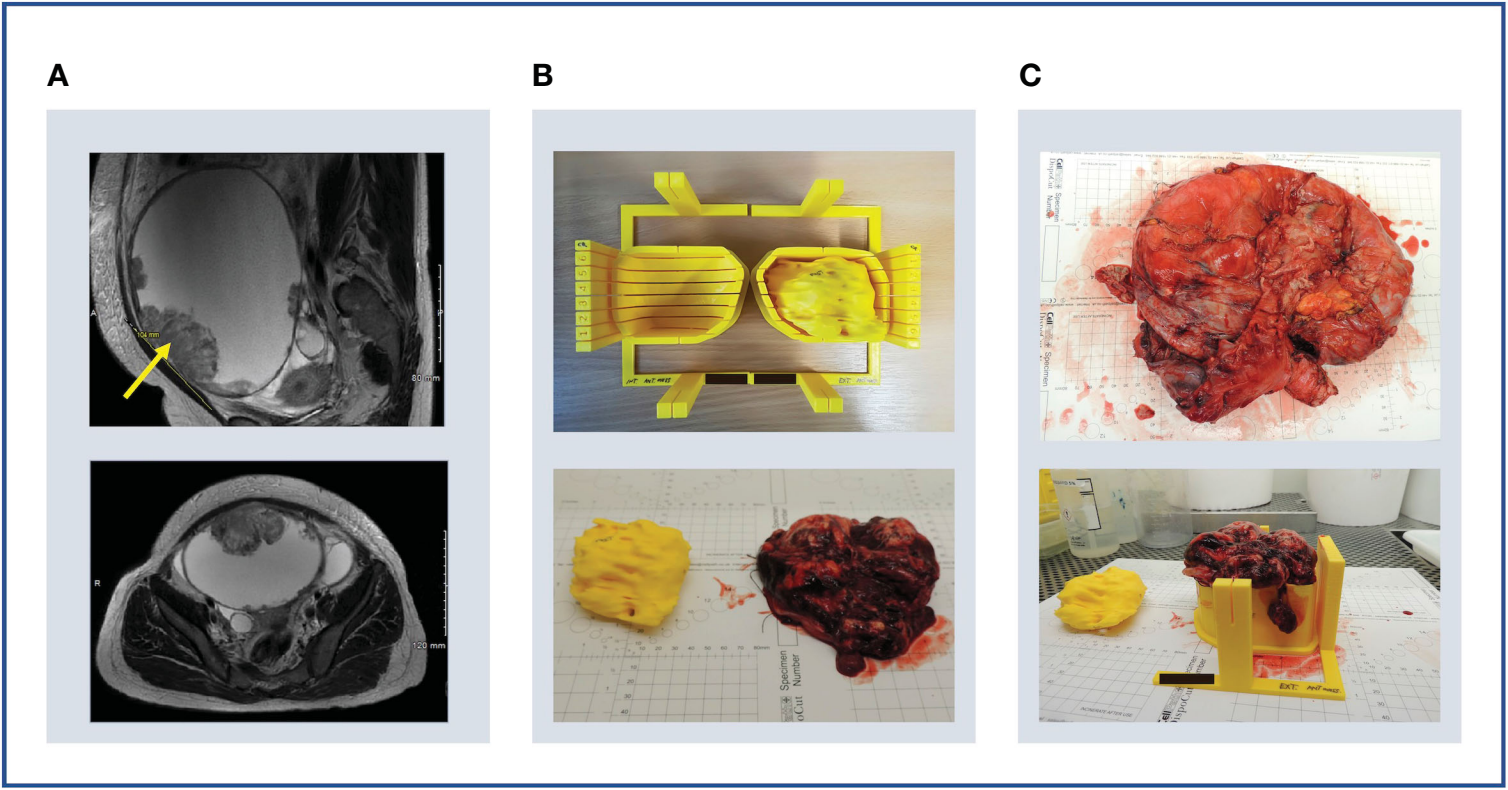
Case 4. **(A)** Upper panel: sagittal MRI image showing large cystic lesion with solid component in anterior wall (arrow). Lower panel: axial MRI image showing the same lesion (arrow) and positions of external (blue) and internal (green) mould bases. Note CT imaging was used for segmentation (MRI images used here for illustrative purposes only). **(B)** Upper panel: internal (left) and external (right) moulds with guides (circled) for orientation cut (dotted line). Lower panel: tumour replica and specimen with size discrepancy. **(C)** Upper panel: the ruptured cyst was removed en bloc –two orientation sutures (orange arrow, other suture on underside of specimen) and the resected cervix (blue arrow) were used for specimen orientation. Lower panel: ultimately, specimen size exceeded predicted mould dimensions due to an unusual interval of 53 days between imaging and surgery during which the tumour had increased in size.

This was an illustrative case of mixed cystic and solid composition often encountered with ovarian tumours (**Figure 10A**). As the main solid lesion of interest was located adjoining the wall of a very large (233×181×136 mm) cyst which would collapse on slicing, the solid part alone was segmented. Two alternative locations for the mould base were also marked during the segmentation step to enable creation of two alternative moulds to hold this solid component – one each for the external and internal projections of the solid mass in relation to the cyst (**Figure 10A**). This accounted for preoperative unpredictability regarding the optimal specimen orientation to preserve tissue integrity and stability for this case.

The mould cavity algorithm was modified to allow for the extension of the mould cavity height beyond the point of maximum tumour width to reduce tissue slice overspill as discussed for Cases 2 and 3. Nonetheless, while the resection specimen resembled the tumour replica in shape and contour, the size exceeded the mould dimensions (**Figures 10B, C**). This reflects the longer time elapsed between the CT imaging used for segmentation and IPS due to individual clinical circumstances (**Table 2**). Meanwhile, the transformation to WCS was successful for this case, demonstrating the utility of adding the ROI of the optimal base of the mould location during the segmentation step.

#### 3.3.5 Case 5

As a final proof of concept of the technical pipeline, we selected a typical HGSOC case with low-volume disease following response to NACT.

The bilateral lesions were segmented on MRI which was performed closer to the time of surgery than CT by 21 days (see Section 3.2). From a technical perspective, the transition from CT to MRI was seamless and did not require any alterations to the pipeline, as the inputs from both modalities are a DICOM set of images and a DICOM-RT file containing the segmented ROIs.

Both lesions were of small size and approximately ovoidal in shape, with their longest axis along the craniocaudal axis (both below 4 cm). This meant that only a limited number of slices with a high thickness-to-diameter ratio would result from using the original slice thickness of 10mm, which would be unideal for multisampling within tissue slices. To enable the option of increasing tissue sampling resolution, the impact of reducing the intervals between slits was explored in preparation for Case 5. A test mould of the previous case was re-modelled and 3D-printed with reduced slice thickness from 10 mm to 5 mm, which confirmed that mould sturdiness was retained.

**Figure 11:**
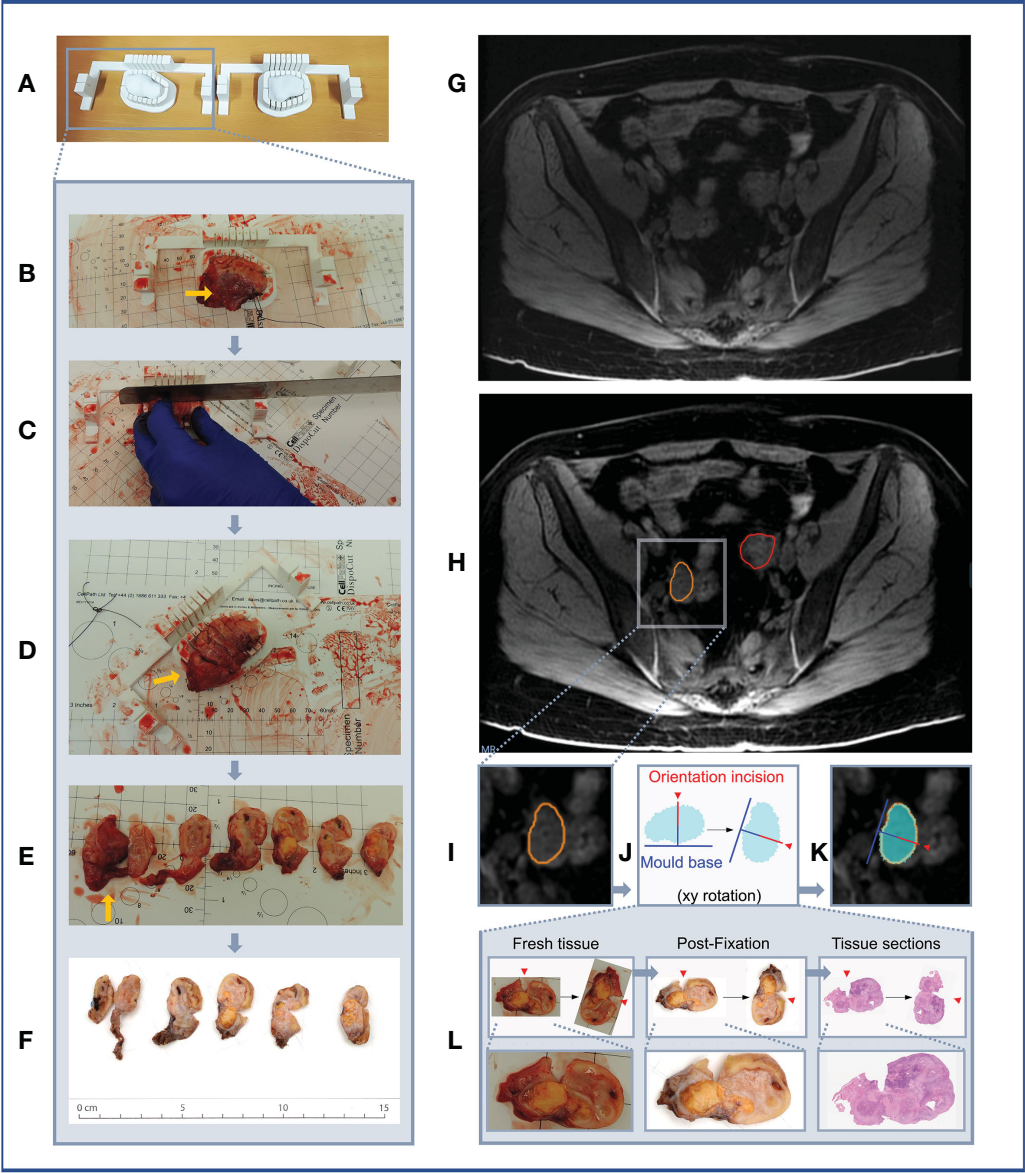
Case 5. **(A–F)** Demonstration of 3D mould-based histological processing workflow, with the right ovary as a representative example. Yellow arrow: Fallopian Tube. **(G–L)** Overview of co-registration between radiology and tissues. **(A)** Right and left moulds and corresponding tumour replicas. **(B)** Right ovary placed within corresponding mould. **(C)** Partial orientation incision placed across specimen using in-built orientation guide. **(D)** Specimen sliced within mould. **(E)** Fresh 5mm thickness tissue slices resulting from slicing within mould. Notches from orientation incision allow slices to be orientated in relation to each other. **(F)** Tissue slices following formalin fixation. **(G)** MRI image. **(H)** MRI image with bilateral ovarian segmentations overlayed (orange: right ovary, red: left ovary). **(I)** Magnified image of segmented right ovary. **(J)** Computationally derived expected outline of the corresponding tissue slice and mould base position, rotated on the xy plane to correspond to the base position specified at the segmentation step. The red arrow and segment represent the angle and depth of the in-built orientation incision. **(K)** Right ovarian segmentation with tissue outline and mould base overlayed. **(L)** The orientation incision allowed tissue lice orientation to be preserved across processing steps, from the fresh, fixed to sectioned tissue stages (haematoxylin and eosin stained section shown).

The two moulds and corresponding tumour replicas for this case were successfully printed in time for the resection procedure (**Figure 11A**). Structural robustness of the final moulds was maintained with the updated slice thickness of 5 mm, and these slits were successfully used for specimen slicing for both lesions (**Figures 11B–E**). Whilst tumour cellularity was below the fresh tissue sampling threshold, the reduced slice thickness allowed all slices from each ovary to be placed in tissue cassettes for processing into FFPE blocks. The orientation cut was essential in enabling orientation of tissue sections resulting from these FFPE blocks **Figures 11F, L**.

### 3.4 Prospective research pathway development

In addition to providing proof of concept of 3D mould-based multi-sampling in a variety of ovarian lesions, this pilot study sought to test the feasibility of prospective implementation of the method in a busy clinical setting at a tertiary hospital. With patient care remaining the central priority throughout, clinical workflow constraints dictated the following absolute requirements for an ovarian 3D mould research pathway (1): to be compatible with standard clinical procedures without any interference or delay to patients’ standard of care treatment pathways (2); to be able to adapt to a variety of real-world timelines, including short treatment planning intervals for cases scheduled for elective surgery on an urgent basis (3); to have no or negligible impact on clinical workload (4); to maximise efficiency and effectiveness of communication between clinical and research team members from diverse disciplines.

**Figure 12:**
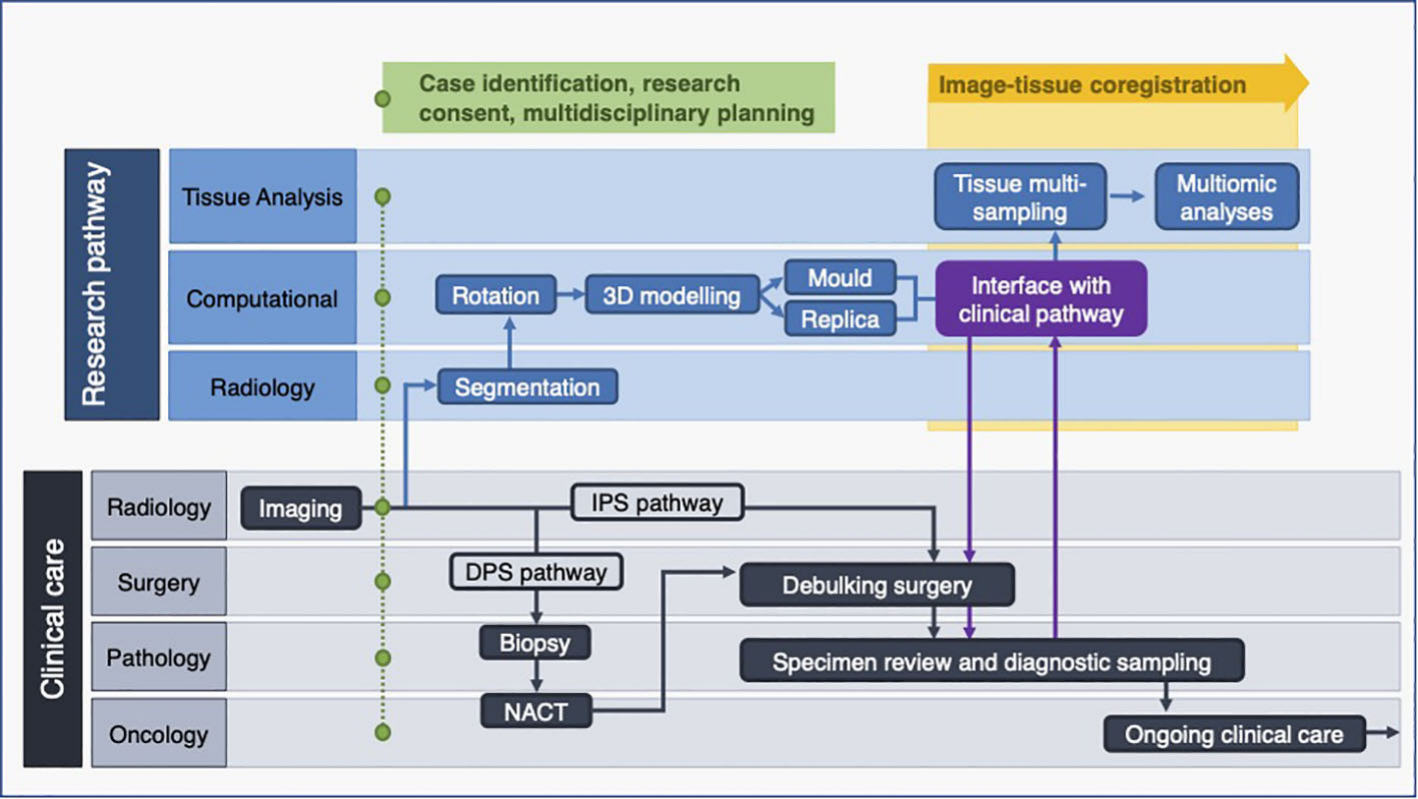
Multidisciplinary research pathway for malignant pelvic ovarian tumours. Interface with clinical pathway shown in purple. DPS, (delayed primary surgery); IPS, (immediate primary surgery); NACT, (neoadjuvant chemotherapy).

To achieve this, we developed a research pathway that fits entirely *around* the standard clinical care pathway for patients with suspected or confirmed ovarian cancer (**Figure 12**). Following case identification and research consent by a member of our research team, potential cases were selected *via* rapid case review between the Research Radiology team and key members of each research discipline. A designated clinical research fellow (MR: Medical Oncologist in training) coordinated case selection and all communication with clinical teams as a single point of contact, disseminating required case information and confirming availability for participation by each team. Case confirmation triggered the research pathway which interfaces with the clinical care pathway across two phases: from the point of specimen retrieval intraoperatively through to tissue sampling in Histopathology, both coordinated by the same clinical fellow for consistency. The surgical and histopathology teams were briefed at their convenience on the day of surgery with no additional workload or delay.

**Figure 13:**
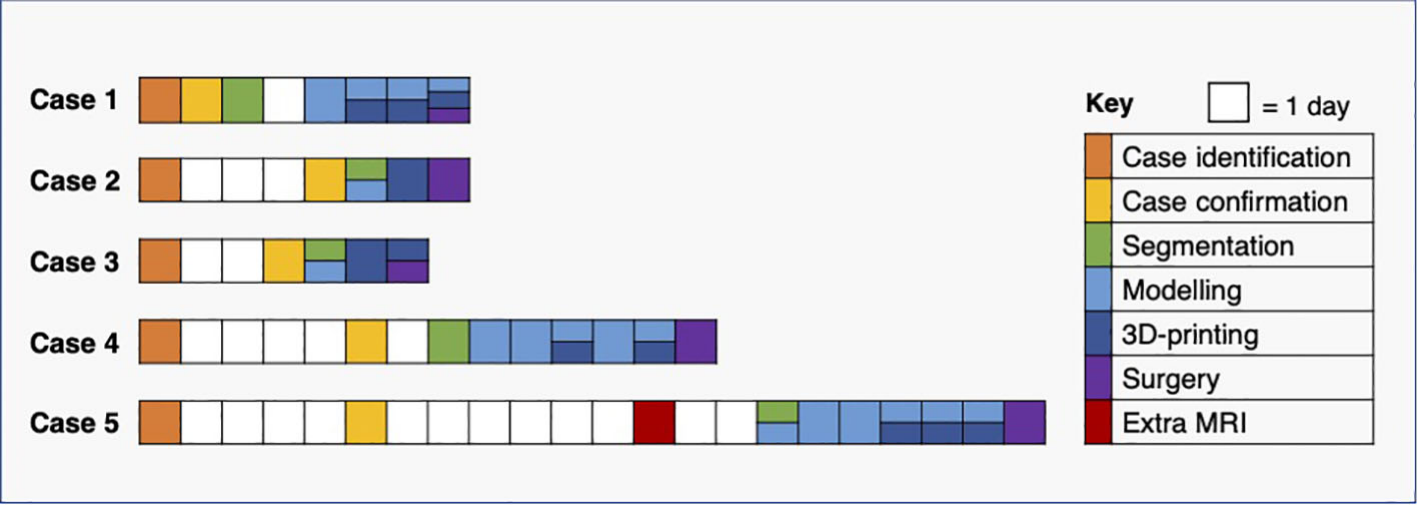
Pathway timelines for each case. All baseline CT imaging took place prior to case identification (see **Figure 12**).

The interval between case confirmation and surgery ranged between 3 and 16 days, and the shortest window for segmentation, modelling and 3D-printing was two and a half days. We demonstrated that all elements of the pathway required for producing moulds and tumour replicas in time for surgery could be completed for every clinical timeline encountered in this pilot study (**Figure 13**).

## 4 Discussion

The high levels of heterogeneity at the radiomic, cellular and genomic levels in HGSOC are individually known to carry prognostic significance (24–26), however the spatial relationship between these multiscale features has not been defined. In this work, we developed a computational pipeline for generating lesion-specific 3D-printed moulds to allow for the co-registration of imaging and tissue regions, based directly on insights drawn from a prospective pilot study.

3D moulds have been used in preclinical and translational studies for correlating imaging and tissue-derived data in a number of tumour-types, including ovarian (6), prostate (9–16), hepatic (17, 18) and renal cancers (7, 19). However, no previous study has investigated a variety of ovarian lesion characteristics seen in practice to generate a pipeline that caters to this diversity. Furthermore, most published works to date have presented a final method, without detailing intermediate technical insights relating to both successful and unsuccessful aspects of the mould development process which could be key to reproducing or building upon these existing pipelines. Given this, we provided a detailed account of our entire iterative process of pipeline development, and highlighted critical disease-specific challenges that should be considered in any future 3D mould-based studies of ovarian tumours and HGSOC in particular.

A key anatomical challenge in achieving accurate image-tissue co-registration in ovarian tumours is the lack of universal anatomical structures that can reliably be utilised for computational tumour rotation and specimen orientation. This contrasts with other tumour types for which anatomical contexts are inherently preserved post-resection, as exemplified by the use of the tumour-hilum contact point of radical nephrectomy specimens in determining mould base position and confirming correct specimen positioning (7). We demonstrated that the utility of en bloc resection for orientation is limited in ovarian tumours (**Case 1, Supplementary Material**), particularly given the highly tortuous and mobile nature of the Fallopian tube, as well as the separation of ovarian tumours from adjoining structures prior to slicing during standard histopathological tissue processing. Instead, we addressed this through two innovations. Firstly, we achieved automated tumour rotation by introducing an additional annotation to mark optimal base of the mould location at the segmentation stage. This had the advantage of allowing the slicing to be constrained to an anatomical plane of choice. Secondly, by creating 3D-printed tumour replicas, we were able to conduct visual comparison of predicted and actual resection specimen characteristics and guide correct specimen placement in the mould.

From a clinical perspective, the main challenge in implementing 3D mould-based sampling of resection specimens in HGSOC is that an increasing proportion of patients are treated with NACT followed by DPS (27). While the majority of HGSOC cases are diagnosed at an advanced stage and often with large-volume disease (28), tumours resected at DPS in the context of NACT response can be of markedly small volume. The volume of the lesions was of particular relevance during computational tumour modelling, as meshing and smoothing processes have greater effects on small volumes. The use of tumour replicas was thereby key in confirming that the modelled tumour volumes accurately resembled the shape and size of the resected specimen for six out of the seven lesions, including those below 15 cm^3^. Unfortunately, given the scope and size (five cases, seven lesions) of the pilot study, we could not establish a meaningful volumetric tolerance to quantitatively study the suitability of a lesion for our mould design. However, other lesion characteristics beyond volume are crucial for the mould design. For instance, the length of the tumour in the axial plane (slicing direction) highlights the importance of the presence of tunable parameters including slice thickness, in order to allow the granularity of tissue sampling to be adjusted on a case-by-case basis and ensure that a sufficient number of slices can be acquired for meaningful tissue multisampling. We have shown that the structural integrity of moulds was retained when halving slice thickness from 10 mm to 5 mm while increasing knife slits from 0.5 mm to 1 mm. This was of particular importance in Case 5, in which the post-NACT tumour was found to have negligible tumour cellularity below the threshold required for fresh tissue-based genomic analysis. By reducing the slice thickness, all slices could be placed in standard tissue cassettes to be made into FFPE blocks, which can instead be sectioned and subjected to other analyses such as computational pathology approaches.

Accurate image-tissue co-registration relies on two further technical properties of mould design, namely stability and orientation of tissue slices while within the mould. Firstly, as shown by Cases 2 and 3, the accuracy of specimen slicing is dependent on the stability and support afforded by its mould, which requires both the robustness of the mould itself, as well as a sufficient mould height to provide lateral support. To avoid specimen overspill, we therefore do not constrain mould height to the point at which the widest xy dimension of the tumour occurs (7), but rather allow the percentage height covered by the mould cavity to be tunable according to case-specific requirements. Specimen stability is also dependent on the structural composition across the specimen itself, which may not be possible to determine preoperatively, particularly based on CT imaging alone. The ability to determine mould base location during segmentation, and to specify multiple options in order to 3D-print alternative moulds in advance of surgery, is therefore a key strength that contributes to the flexibility of our pipeline, as confirmed by Case 4.

Secondly, without a method to mark tumour slices within the mould, their orientation to each other and to the radiological imaging is immediately lost upon removal from the mould, particularly for ovoid tissues lacking obvious shape or substructure characteristics (**Figure 9**). Our in-built partial-thickness perpendicular orientation slit is therefore a powerful improvement on existing co-registration pipelines which allows slice orientation to be maintained both before and after fixation and embedding, but crucially is simple and rapid enough to be implemented in a clinical setting (**Figure 11**). To perform co-registration, we generated slice-by-slice outlines of tissue slices expected from each slicing position in the mould (**Figure 11J**). These outlines include the positions of the mould base and orientation incision (the depth of this partial incision is marked in red in **Figure 11J**), such that the physical orientation incisions of tissue slices can be used to orient them against the slice-by-slice outlines. A critical mould design feature here is that the tissue slicing corresponds to axial slices in the segmented radiological imaging, meaning that each slice outline and corresponding tissue slice simply requires a single rotation by a fixed angle along the xy plane to be aligned with the mould base position as specified at the image segmentation step (**Figures 11G–K**). Given the persisting physical presence of the orientation incision, this orientation process can be undertaken at the fresh or post-fixation stages of tissue processing. In our final case, we were able to extend this co-registration approach to the subsequent whole-slide histological sections generated from the FFPE blocks, given the context of low-volume residual disease post-NACT where the 5mm-thickness tissue sections could be embedded in full within standard tissue cassettes (**Figure 11L**). The ability to perform coregistered sampling before or after sample fixation allows an important level of flexibility in being able to adapt tissue sampling to post-operative findings – for instance, when frozen section review of the fresh specimen in Case 5 demonstrated insufficient histopathological tumour cellularity for genomic analysis by taking fresh tissue biopsies, it was possible to make a rapid real-time decision to prioritise whole-slice analyses of FFPE blocks at the next stage of routine clinical tissue processing.

In practice, a research pathway of this kind must be highly adaptable to the requirements of a patient’s individual treatment pathway and tumour characteristics, and be implemented efficiently with minimal to no impact on resources and professional workloads within the wider healthcare setting. While previous work has used multiparametric MRI and PET/CT (6, 29), we were able to successfully implement our framework based on more routinely available CT (N = 4), as well as MRI (N = 1). In addition to addressing the aforementioned technical and anatomical challenges by implementing a range of tunable parameters, our research pipeline was able to accommodate further clinical scenarios including mould generation for bilateral disease (Cases 3 and 5) and for the solid component of a large mixed solid-cystic mass (Case 4). All research components were completed in time for each patient’s surgical procedure without interference with individual treatment timelines, with a minimum interval of three days between case selection and debulking surgery. Focused and coordinated communication between multidisciplinary clinical professionals from the Radiology, Surgery, Oncology and Histopathology departments was key in enabling timely research pathway implementation.

Our approach is based on clinical standard-of-care imaging and did not include any dedicated imaging sessions for optimising mould design. This was the likely reason for the mismatch between the segmented tumour and actual specimen volume in Case 4, who had the longest elapsed time between imaging and surgery. Of note, in most routine clinical settings, cross-sectional imaging is undertaken in prior preparation for but not on the day of surgery, and the cost of further imaging or risk of radiation exposure is not justified without unique clinical indications. An additional scan, ideally an MRI including T2-weighted images, close to the day of surgery could easily solve this problem in cases with a long delay between the last preoperative standard-of-care scan and surgery. However, we found that all other segmentations generated tumour replicas which closely resembled the resected specimen, including that of a post-NACT case whose imaging was performed 40 days prior to surgery.

A second limitation is that these pilot cases did not yield HGSOC tissue for multi-site genomic profiling. This highlights the inherent challenges of case selection in the context of standard treatment pathways for advanced pelvic ovarian tumours. For cases undergoing IPS, tumours are likely to be of greater volume and cellularity for sampling, but histological diagnosis is often lacking preoperatively. An IPS case may be benign despite significantly elevated CA-125 and suspicious qualitative radiological features (as we found with Cases 2 and 3), however a significant proportion of tumour will be required for diagnostic purposes and may leave a small proportion for research purposes even if malignancy is confirmed. DPS cases are more likely to have limited remaining tumour tissue for sampling (Case 5), or have greater cystic components with or without sufficient tissue for sampling as a feature of NACT response (Case 1). With regard to tumours containing cystic components, we have shown that moulds can be modelled for a discrete solid component, and this can be extended to multiple distinct areas of solid disease. The majority of cases for which tumours are predominantly cystic with minimal or no residual tumour tissue after NACT will be identifiable at the preoperative imaging stage. The manual base placement step allows the base to be placed by the surface with the largest proportion of solid components to maximise stability – however specimen stability would remain challenging in very complex masses containing a large number of discrete cystic and solid areas. Future case selection will be informed by these clinical factors, and may benefit from focusing on IPS cases with prior tissue diagnosis from laparoscopic biopsy or DPS cases with predominantly solid residual disease post-NACT.

This work was motivated by the need to guide comprehensive multi-sampling of pelvic tumour resected specimens for correlation of imaging and genomic features. A key strength of our work is that the pipeline facilitates direct co-registration of tissue coordinates and imaging slices on the same transverse axis by constraining tissue slicing to the axial plane. Our pipeline is thereby highly suited to implementing systematic grid-based sampling, by aligning a grid-based system with the orientation cut of each tissue slice in order to directly map sample coordinates onto spatial coordinates of the corresponding image slice. Such a coordinate-based system will be well-suited to larger-scale prospective biological validation of previously described radiomic ‘habitats’ (6).

## 5 Conclusion

We developed a computational pipeline for modelling lesion-specific 3D-printed moulds to guide slicing and multi-sampling of solid pelvic tumour resection specimens. This work provides a framework for obtaining spatially co-registered imaging and multi-sampled tissue data, thus aiming to perform detailed multi-level characterisation of intratumoural heterogeneity of pelvic gynaecological tumours. We highlight specific challenges pertaining to ovarian tumours, propose a highly tunable design that is adaptable to the specific requirements of a given case, and provide recommendations for pathway implementation. This pipeline can be implemented alongside clinical treatment pathways for patients with newly diagnosed HGSOC and other ovarian tumours.

## Data availability statement

The datasets presented in this article are not readily available because of ethical restrictions due to the presence of patient data. Requests to access the datasets should be directed to Prof Evis Sala, es220@medschl.cam.ac.uk.

## Ethics statement

The studies involving human participants were reviewed and approved by Cambridgeshire and Hertfordshire Research Ethics Committee approval reference 08/H0306/61. The patients/participants provided their written informed consent to participate in this study.

## Author contributions

Conceptualisation: MR, MD-O, CM, VB, RW, LR, MJ-L, MC-O, JB, LES and ES. Methodology: MR, MD-O, CM, VB, RW, LR, MJ-L, MC-O, JB, LES and ES. Software: MD-O (original for this work), AG, MG and MC-O (previous developments). Validation: MD-O, MR, CM, VB, RW, LR, MJ-L, MC-O, LES and ES. Investigation: MR, MD-O, CM, VB, LES, RW, MJ-L, JB and ES. Resources: HB, KH, PP, MJ-L, JB and ES. Data curation: MD-O, MR and LES. Visualisation: MR, MD-O and CM. Writing—original draft: MD-O and MR. Writing—review and editing: MR, MD-O, LES, CM, VB, RW, LR, AG, MJ-L, MG, SU, HB, KH, PP, MC-O, JDB and ES. Supervision: ES, LES, MC-O, JB and RW. Project administration: MR, JB and ES. Funding acquisition: JB and ES. All authors contributed to the article and approved the submitted version.
