# Supplementary material for: Lesion-specific 3D-printed moulds for image-guided tissue multi-sampling of ovarian tumours: A prospective pilot study
Source: Front Oncol. 2023 Feb 13;13:1085874. doi: 10.3389/fonc.2023.1085874 (PMC9969130; doi:10.3389/fonc.2023.1085874)
Supplement: Supplementary file 1 [file DataSheet_1.docx]

Supplementary Material

Lesion-Specific 3D-Printed Moulds for Image-Guided Tissue Multi-Sampling of Ovarian Tumours: a prospective pilot study

Maria Delgado-Ortet^†^, Marika A.V. Reinius^†^, Cathal McCague, Vlad Bura, Ramona Woitek, Leonardo Rundo, Andrew B. Gill, Marcel Gehrung, Stephan Ursprung, Helen Bolton, Krishnayan Haldar, Pubudu Pathiraja, James D. Brenton, Mireia Crispin-Ortuzar, Mercedes Jimenez-Linan, Lorena Escudero Sanchez, Evis Sala^*^

**^†^** These authors contributed equally to this work and share first authorship.

*** Correspondence:**Prof Evis Sala, MD, PhD, FRCR
es220@medschl.cam.ac.uk

# Two alternative moulds for Case 1

Two moulds were printed: one lesion-specific (left ovarian tumour only) mould (**Figure 5A**), and one mould accommodating the whole en bloc specimen including the tumour, uterus, contralateral ovary and bilateral Fallopian tubes (**Supplementary Figure 1**). The purpose of the latter was to test whether adjacent structures may serve as anatomical landmarks for pelvic specimen orientation. However, the lack of fixed spatial relationships between the ovaries, Fallopian tubes and uterus renders successful recapitulation of the *in vivo* spatial relationships between these in an en bloc mould very challenging. Meanwhile, lesion-specific mould design allowed for a closer specimen fit and favourable interface with standard histopathological workflows for ovarian lesions, which are commonly removed from adjacent structures before slicing separately, if not already separated intraoperatively. Given these observations, the lesion-specific approach was applied in all cases.


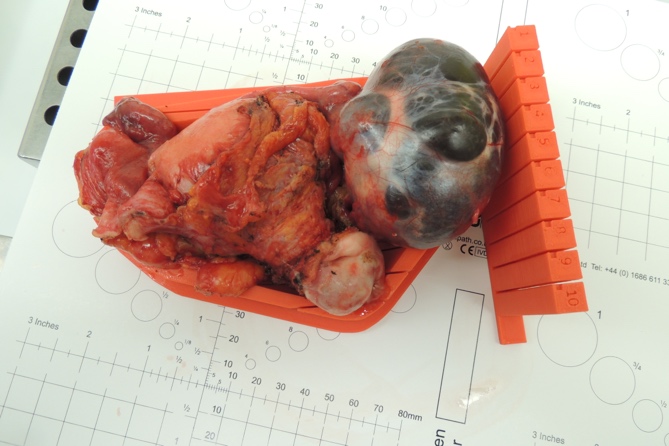


**Supplementary Figure 1.** Specimen placed in en bloc mould version.

# Supplementary Tables

**Supplementary Table 1.** CT acquisition parameters for the preoperative scans used for segmentation in Cases 1-4.

|  | Case 1 | Case 2 | Case 3 | Case 4 |
| --- | --- | --- | --- | --- |
| Vendor | SIEMENS | SIEMENS | SIEMENS | TOSHIBA |
| Model | SOMATOM Definition AS | SOMATOM Definition Edge | SOMATOM Definition AS | Aquilion PRIME |
| Reconstruction kernel | I26f3 | Br36f3 | I26f3 | FC11 |
| Reconstruction diameter [mm] | 368 | 370 | 350 | 414.062 |
| KVP | 100 | 100 | 100 | 120 |
| Exposure [mA] | 164 | 245 | 206 | 90 |
| Matrix size [pixels] | 512 × 512 × 293 | 512 × 512 × 270 | 512 × 512 × 66 | 512 × 512 × 638 |
| Slice thickness [mm] | 2 | 2 | 2 | 1 |
| Pixel width [mm] | [0.71875, 0.71875] | [0.72265625, 0.72265625] | [0.68359375, 0.68359375] | [0.808, 0.808] |

**Supplementary Table 2.** MRI acquisition parameters for the preoperative scan used for segmentation in Case 5.

|  | Case 5 |
| --- | --- |
| Vendor | GE Medical Systems |
| Model | SIGNA PET/MR |
| Magnetic Field Strength | 3 T |
| Sequence | LAVA-Flex (First Gradient Echo) |
| Repetition Time (ms) | 4.372 |
| Echo Time (ms) | 1.846 |
| Flip Angle | 12 degrees |
| Acquired matrix [pixels] | 256 × 224 |
| Matrix size [pixels] | 512 × 512 × 72 |
| Slice thickness [mm] | 5 |
| Spacing between slices [mm] | 2.5 |
| Pixel width [mm] | [0.6641, 0.6641] |
